# A Promotive Process of Resource Gain Against Harsh and Inconsistent Discipline in Mothers Coping With Breast Cancer: A Serial Mediation Model

**DOI:** 10.3389/fpsyt.2022.859604

**Published:** 2022-06-16

**Authors:** Osnat Zamir, Gabriella Bentley, Yaliu He

**Affiliations:** ^1^The Paul Baerwald School of Social Work and Social Welfare, Hebrew University of Jerusalem, Jerusalem, Israel; ^2^Social Work & Marriage and Family Therapy Department, Iona College, New Rochelle, NY, United States

**Keywords:** resource gain, income, paternal involvement, post-traumatic symptoms, maternal parenting practices

## Abstract

Breast cancer is a life-threatening disease and a source of enduring stress. The Family Stress Model posits that psychological distress provoked by stressful conditions may spill over and intensify harsh and inconsistent parental discipline. However, the Conservation of Resources theory posits that having more resources may lead to further resource gain, which may promote adaptive coping with adversities. Therefore, this study examined a serial mediation model in which financial resources (income) are predicted to be associated with more interpersonal resources (paternal involvement). The latter is expected to be linked with less maternal post-traumatic stress symptoms, which, in turn, should be associated with less harsh and inconsistent discipline in mothers coping with breast cancer. A sample of 100 Israeli mothers receiving breast cancer treatments was recruited through social media. The participants completed online self-report questionnaires. Structural Equation Modeling indicated significant serial mediation, in which a greater income level was associated with more paternal involvement, which was linked to a lower level of maternal post-traumatic symptoms. The latter, in turn, was associated with less harsh and inconsistent maternal discipline practices. We controlled for illness severity and the time since diagnosis, which did not predict maternal discipline practices. The study suggests that although breast cancer is a stressful condition for any family, having more financial resources can be a significant promotive factor predicting a cascading process by which paternal functioning facilitates better mental health of mothers, and, in turn, better maternal parenting practices.

## Introduction

Breast cancer (BC) has been identified as the most common cancer and the most frequent cause of cancer mortality in women. In 2020, about 2.3 million BC diagnoses and 685,000 deaths were documented worldwide. BC is more prevalent in older ages (1), but some women may face it at a young age (2). Young women are more likely to have at least one minor child living at home (3), and are more willing than non-parent patients to receive aggressive treatments to increase their likelihood of survival (4, 5). BC patients with dependent children cope not only with intensive treatments [i.e., surgery, radiation therapy, endocrine therapy, chemotherapy, or targeted biologic therapy; (6)] and their side effects (e.g., pain, fatigue) but also with childcare (7). Because mothers are typically the primary caregivers, they experience more parenting concerns than fathers who are cancer patients (8). Mothers with BC report a range of problems related to parenting, including physical limitations (e.g., fatigue) and difficulty in maintaining the child's routine [for a review, see (9)]. They also report lower parental self-efficacy and satisfaction compared with healthy mothers (10, 11). Moreover, their children tend to experience more emotional distress, externalizing, and internalizing behavioral problems compared with children of non-ill mothers (12, 13).

Long-standing research emphasizes that parenting is a major protective factor for healthy child development in the face of adversity (14). For example, while illness characteristics (e.g., cancer site, stage, time since diagnosis) were not found to be associated with the physical, emotional, and social wellbeing of children whose parents had cancer (7), supportive caregiving was associated with lower child anxiety levels (15). It is therefore imperative to identify promotive processes (e.g., resources gain) for maternal parenting practices while coping with BC, which is the focus of the current study. Specifically, we tested whether financial resources are indirectly associated with less harsh and inconstant maternal discipline through a serial mediation process involving greater paternal involvement, which in turn is linked with less maternal post-traumatic stress symptoms (PTSS). The findings of this study may facilitate the development of interventions designed to improve parenting practices for mothers with BC, which may ultimately benefit their children's adjustment.

### A Theoretical Framework

The Family Stress Model (FSM) posits that stressful conditions may intensify emotional distress, which may deplete the psychological resources of parents, making them more inclined to use harsh or inconsistent disciplinary practices (16, 17). Harsh discipline is defined as coercive acts directed toward children, expressed by verbal (e.g., yelling) or physical aggression (18). Inconsistent discipline refers to a lack of adherence to rules and standards related to children's behavior (19). Such negative parent-child interchanges were postulated to result in behavioral problems in children (16, 17). To date, research on the parenting quality of cancer patients has focused mostly on communication about the disease and empathic responses to children [for a review see (12)], but not much is known about harsh and inconsistent discipline practices while undergoing BC treatments.

The FSM was supported in several stressful conditions, including economic hardship (16, 17, 20) and traumatic stress (21–23). BC is a life-threatening disease, which may trigger traumatic psychological reactions (24). Cancer patients may experience fear of cancer recurrence and fear of death (25), and they are at risk for post-traumatic stress disorder [PTSD; for reviews see (26, 27)], even more than other types of cancer [for a review see (28)]. The prevalence of PTSD in BC patients varies from 3 to 32.3% across studies, and it is more prevalent at younger ages (25, 29). Although PTSS have been linked with more harsh and inconsistent discipline practices (23, 30), it was not studied in the context of BC. The research on BC patients shows that greater psychological distress is associated with more difficulties in parental (11, 31) and family functioning (32). Hence, the current study aims to expand the scope of the literature by focusing on the associations between PTSS and harsh and inconsistent discipline in mothers dealing with BC treatments.

### The Promotive Role of Resource Gain When Facing BC

The FSM posits that even under stressful conditions, promotive factors (i.e., factors that directly predict positive outcomes) may facilitate better psychological and interpersonal functioning of family members (33). The Conservation of Resources theory [COR; (34)] further illuminates a mechanism by which promotive factors operate to enhance better adaptation to trauma. Specifically, in the wake of trauma, individuals seek to secure, retain, and gain resources, but they often lose them, which may lead to further loss of resources (35). Personal (e.g., personality traits), social (e.g., support), and material resources (or lack thereof) determine the extent to which a situation is perceived as stressful vs. manageable (36). Thus, a major loss of crucial resources may lead to a traumatic response (35). In contrast, having more resources may lead to a spiraling process of resource gain (36), which provides a sense of security, a basic foundation for one's survival, and thus strengthens the psychological resilience for traumatic stress (34). Relying on the COR theory, we were interested in examining a promotive process of resource gain in mothers coping with BC. Specifically, we aimed to examine whether their material resources (i.e., income) are associated with more social resources (i.e., paternal involvement), which in turn predict lower PTSS and sequentially less harsh and inconsistent discipline.

### Income as a Promotive Resource for Families Facing BC

Income is a vital resource for family functioning and more so when dealing with illness, a situation that often requires paying for costly medical, psychological, or instrumental services (37, 38). Hence, besides health concerns and demands, families dealing with BC may face economic strains (39). The FSM posits that under economic strains, psychological resources may dampen, resulting in disrupted co-parenting relations (16). Indeed, economic strains have been negatively associated with co-parenting quality (40). Fathers are particularly sensitive to financial problems, which may impair their co-parenting relationships (41). In this study, we focused on the association between economic resources and paternal involvement. Paternal involvement is defined as cooperative co-parenting, specifically by support provision in caregiving tasks (42), and it is interrelated with co-parenting relations (43).

While economic stress may hinder co-parenting relations through psychological distress (16), the COR theory posits that having more financial resources can help parents cope better with stressors by providing a greater sense of security that facilitates psychological adaptation (34, 36). In fact, parents with greater resources such as higher income tend to invest more time in childrearing and co-parenting activities than those with fewer resources (44). As such, it could be that when dealing with BC, more economic resources may help fathers be more engaged in paternal tasks.

The amount of support provided by fathers with wives diagnosed with BC tends to depend on their partner's level of physical impairment (45). While coping with their partner's cancer, fathers often experience strains related to their caregiving roles while still handling other tasks of work and finance (46, 47). Research on BC indicates that more frequent BC demands are associated with greater depressive mood, which is associated with lower marital quality (48). However, the effect of financial resources on paternal involvement and their cascading effect on maternal harsh and inconsistent discipline in families facing BC has not been studied.

### Paternal Involvement as a Promotive Factor Against PTSS

When coping with BC, the support provided by a spouse is a major resource that predicts a better quality of life for women (49). In contrast, having an unsupportive or unhelpful partner predicts a higher level of anxiety in women with BC (50). Because cancer treatments may impede maternal functioning (7), and because mothers are often the primary caregivers, paternal involvement can serve as an interpersonal resource. Greater resources are postulated by the COR theory to promote a greater sense of security that facilitates psychological resilience in the face of trauma (34).

Social support has been found to be a protective factor against the development of PTSS following potentially traumatic events (51, 52), such as a cancer diagnosis (25, 53). More social support also predicts greater post-traumatic growth and better quality of life in women survivors of BC (54, 55). Similarly, a higher quality of co-parenting relations predicts lower levels of depressive symptoms and stress in mothers expecting their second child (56). As such, it could be that greater paternal involvement, as a form of support, will be associated with lower PTSS in mothers coping with BC.

The involvement of fathers may lead to less harsh and inconsistent maternal practices troughs lower PTSS levels in mothers. Given that social support and marital relations are associated with better family functioning and higher levels of parenting confidence when coping with cancer (48), and that lower PTSS are associated with lower harsh and inconsistent discipline (23), it could be that greater paternal involvement will be negatively associated with harsh and inconsistent discipline practices in mothers with BC, mediated by lower maternal PTSS levels.

In sum, the FSM postulates that psychological symptoms associated with stress may spill over and increase harsh and inconsistent parenting (16, 17). However, promotive factors may facilitate better psychological and family functioning (33). The COR theory further explains that resources can be accumulated and thereby promote better psychological adjustment to traumatic stress (36). Research evidence supports the FSM in the context of trauma (23), and points to various promotive factors for families facing adversity (33). However, evidence regarding the association between PTSS and harsh and inconsistent discipline in women fighting BC is lacking. Moreover, the promotive role of resource gain for mental and parental resilience in mothers battling BC has not yet been studied. These gaps are addressed in the current study.

### The Current Study

The present study aims to examine a resilience process, by which promotive factors, namely, economic resources, are serially linked to less harsh and inconsistent discipline of mothers coping with BC through more paternal involvement and lower maternal PTSS. Relying on the FSM and COR theory, we tested whether more economic resources (i.e., income level) are associated with more interpersonal resources (i.e., paternal involvement), which are related to lower maternal PTSS, and in turn, less harsh and inconsistent discipline in mothers coping with BC treatments.

### Research Hypotheses

1. Lower family income levels will be associated with more harsh and inconsistent discipline of mothers coping with BC.
2. The association between income level and harsh and inconsistent discipline will be serially mediated by paternal involvement and maternal PTSS levels. Within this hypothesis, we hypothesized that: (a) paternal involvement will mediate the link between income level and maternal PTSS levels, and (b) maternal PTSS levels will mediate the link between paternal involvement and harsh and inconsistent discipline.

## Method

### Participants

The sample included 100 Israeli mothers who have been diagnosed with BC and were undergoing BC treatments. The participants were mothers of children aged 6–17. We focused on this age group because it has been recognized to be at risk for the development of emotional and behavioral problems when parents are dealing with cancer [for a review, see (57)]. The mean age of mothers was 46.02 (*SD* = 6.06). Most of the mothers were married (88%). The rest indicated they are cohabiting with a partner (4%), involved in a relationship (1%), or divorced (7%). Women reported an average of 17.58 years of marriage or current intimate relationship (*SD* = 6.23) and a mean of 2.94 children (*SD* = 1.01). The majority of participants earned at least a Bachelor's degree (66%) and reported an average of 15 years of education (*SD* = 2.75). The majority of the mothers (65.6%) worked at least part-time (68.7%). The vast majority of mothers were born in Israel (90%) and considered themselves secular (67%) and Jewish (99%). All were native Hebrew speakers. The family income of the participants varied between 0 and 5,000 ILS (1%), 5,000–10,000 ILS (18.6%), 10,000–15,000 ILS (20.6%), 15,000–25,000 ILS (41.2%), 25,000–30,000 ILS (15.5%), 30,000 or higher (3.1%). For comparison, the mean family income in Israel in 2018 was 24,872 ILS ($7765) (58).

Most of the mothers reported being diagnosed within the past 12 months (66%). Disease stages were zero (2.1%), one (13.8%), two (37.2%), three (27.7%), or four (19.2%). During the study, mothers reported receiving cancer treatments, including chemotherapy (40%), biological therapy (37%), hormonal therapy (35%), radiation (25%), or other treatments (6%).

### Procedure

After receiving IRB approval from [The Paul Baerwald School of Social Work and Social Welfare at the Hebrew University in Jerusalem], the data were collected between July 2018 to September 2019. Participants were recruited using a convenience sampling method. The first author advertised the study in closed online BC groups on social media. First, mothers who were interested in participating were asked to fill out a short, online screening questionnaire assessing their compatibility with the research criteria. Namely, participants had to be native Hebrew speakers, mothers to children aged 6–17 and diagnosed with BC who are currently undergoing cancer treatments. Mothers who have met the screening criteria were automatically referred to an online survey. Next, mothers consenting to participate were asked to complete an anonymous online self-report questionnaire.

### Measures

#### Income

Mothers were asked to indicate their total family income including salaries or any other income (e.g., social security) by choosing between the following options: 0–5,000 ILS, 5,000–10,000 ILS, 10,000–15,000 ILS, 15,000–25,000 ILS, 25,000–30,000 ILS, 30,000 ILS or higher.

#### Posttraumatic Symptoms

The Hebrew version of the Post-Traumatic Stress Checklist for DSM-5 [PCL-5; (59)] is a 20-item standardized and clinically validated self-report measure assessing PTSD as defined by the DSM-5. We used the Hebrew version of the PCL-C 5, which has been widely used and has shown good psychometric properties [e.g., (60, 61)]. Mothers completed the Hebrew version of the PCL-C (civilian version), in which the items refer to PTSS related to a traumatic experience. Respondents were asked to rate the extent to which they were bothered by each PTSD symptom in the past month using a 5-point scale ranging from 1 (*not at all*) to 5 (*extremely*). Higher sum scores indicate greater PTSS. Cronbach's alphas indicated good internal consistency (α = 0.95).

#### Paternal Involvement

The Hebrew version (62) of the Co-parenting Relationship Scale [CRS; (63)] is a 35-item measure assessing seven co-parenting domains, from which we utilized two subscales reflecting the perceived involvement of fathers in parenting tasks, including (a) the 7-item Endorsement of Partner's Parenting subscale, which assesses the perceived parenting of one's partner (e.g., “My partner is willing to make personal sacrifices to help take care of our child”), and (b) the 2-item Division of Labor subscale, which assesses the perceived division of labor in parenting tasks (e.g., “My partner likes to play with our child and then leave dirty work to me”). Mothers rated each item on a scale ranging from 0 (*not true of us*) to 6 (*very true of us*). A total score of paternal involvement was computed by averaging the items after reversing negatively keyed items (Cronbach's α = 0.87).

#### Harsh and Inconsistent Discipline Practices

The Alabama Parenting Questionnaire [APQ; (64)] is a parent-report measure that includes 42 items assessing five dimensions of parenting practices used on children ages 6-17. We used two subscales assessing harsh and inconsistent discipline practices, including the 6-item Inconsistent Discipline subscale (e.g., “You threaten to punish your child and then you do not actually punish him/her”) and the 3-item Corporal Punishment subscale (e.g., “You yell or scream at your child when he or she has done something wrong”). A total sum score was computed, with higher scores indicating more harsh and inconsistent discipline (Cronbach's α = 0.69). The APQ has demonstrated discriminant and predictive validity (65). The APQ was translated to Hebrew by the first author. The Hebrew version was translated back to English by a scholar not related to this paper and the resulting translation was examined by the first author for accuracy.

#### Socio-Demographic Background

Mothers completed a brief questionnaire assessing socio-demographic factors, including the age of the mother, country of birth, marital status, economic status, education, religious affiliation, health status, number of children, and number of years married or cohabiting.

#### Disease and Treatment

Mothers were asked to report whether they were diagnosed with BC (0 = *No*, 1 = *Yes*), the stage of the disease (0–4), the time since diagnosis (1 = *up to 12 months*, 2 = *more than a year*), and current treatments, including chemotherapy, biological therapy, hormonal therapy, radiation, or other treatments.

### Data Analysis Plan

A preliminary analysis was conducted using SPSS 25. We then specified a path model using structural equation modeling (SEM) *via* Amos 25 (66) to test the serial mediation model. Missing values occurred across variables and participants, but Little's Missing Completely at Random (MCAR) test indicated that the data were missing at random [χ^2^(33) = 33.76, *p* = 0.43]. We, therefore, tested the model using Full Information Maximum Likelihood (FIML) estimation, which uses all available information from the observed data to generate parameter estimates (67). Indirect effects were assessed using Bayesian estimation (68). In this approach, confidence intervals are calculated based on the posterior distribution of the indirect effect as obtained through computerized simulation [Markov Chain Monte Carlo—MCMC; (69)]. When zero falls outside of the 95% confidence interval, it indicates a significant indirect effect.

Power analysis was conducted using Monte Carlo simulation with 1,000 repetitions in Mplus version 8.5. (70). The simulation indicated that we had sufficient power to detect the paths constructing the indirect effect (ranging from 0.81 to 0.99). The power to detect the serial indirect effect with bootstrap confidence intervals (k = 5,000) was 0.79.

## Results

Descriptive statistics and zero-order correlations for the study variables are presented in Table 1. Harsh and inconsistent discipline was negatively associated with income level and paternal involvement but positively associated with PTSS. Income level was positively associated with paternal involvement and negatively associated with PTSS of mothers. Paternal involvement was negatively associated with PTSS. Lastly, mothers who were diagnosed more than a year before participating in the study had a more advanced (higher) BC stage and reported lower levels of paternal involvement.

**Table 1:** Means, standard deviations, and zero-order correlations of the study variables.

	**1**	**2**	**3**	**4**	**5**	**6**	***M***	***SD***
1. Harsh and inconsistent discipline	–						16.11	3.61
2. Income level	−0.27^**^	–					4.51	1.27
3. Paternal involvement	−0.30^**^	0.43^***^	–				5.00	1.35
4. PTSS	0.45^***^	−0.38^***^	−0.51^***^	–			40.65	16.35
5. Stage	−0.12	0.12	0.09	−0.18	–		3.48	1.02
6. Time since diagnosis	−0.06	0.09	−0.23^*^	0.19	0.46^***^	–		

* *p < 0.05*.

** *p < 0.01*.

*** *p < 0.001*.

We conducted additional preliminary analyses to examine the relations between other demographic variables and the outcome variable, harsh and inconsistent discipline. Pearson correlations computed to examine links with maternal age and the number of children, and Spearman correlations computed to examine links with education level, employment, and marital status, yielded non-significant associations with harsh and inconsistent discipline. Hence, we did not include these demographic variables in our models.

**Figure 1:**
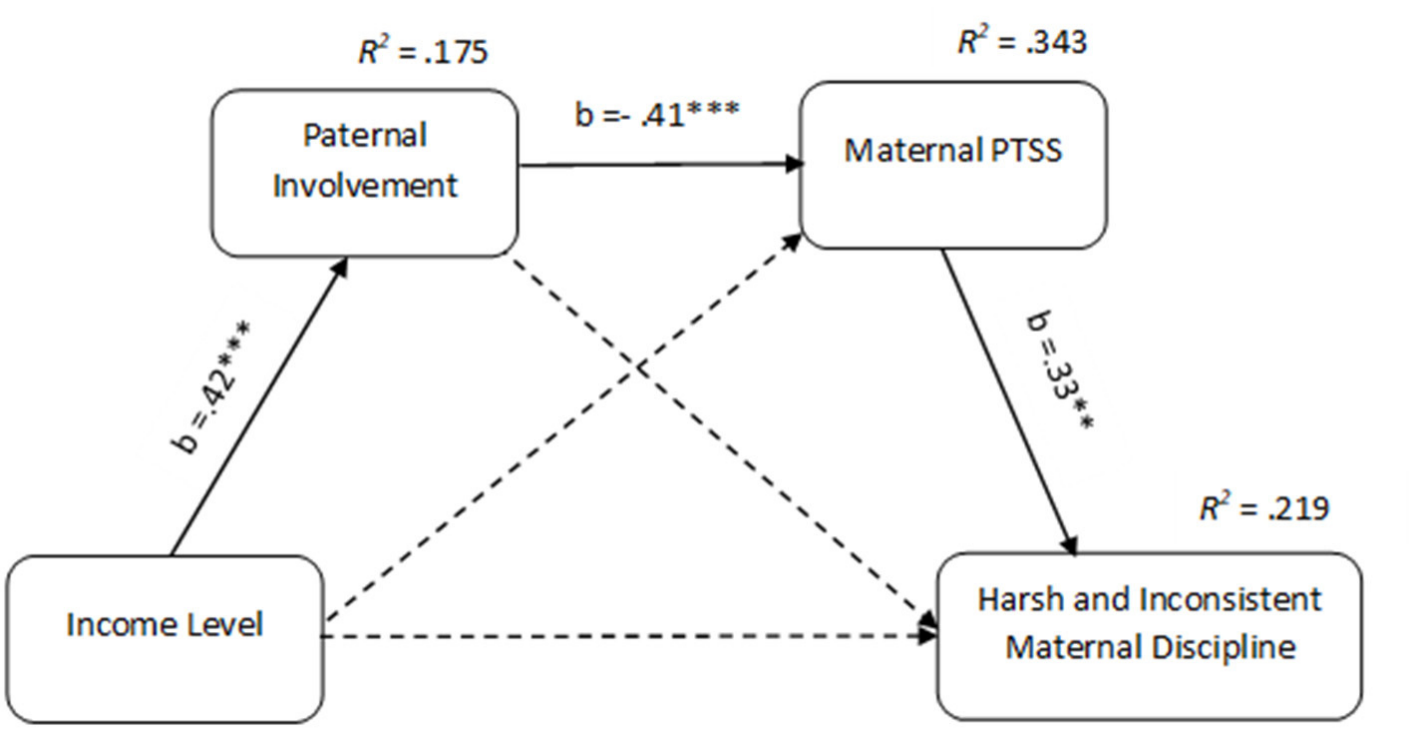
Serial mediation model for the effect of family income on harsh and inconsistent maternal discipline *via* paternal involvement and PTSS of mothers coping with breast cancer. PTSS, Post Traumatic Stress Symptoms. Control variables: cancer stage and time since diagnosis. Entries are standardized structural coefficients. Dashed paths were not found to be significant. ^**^p < 0.01. ^***^p < 0.001.

Next we specified a serial mediation path model in which family income is associated with harsh and inconsistent discipline directly and indirectly through paternal involvement and maternal PTSS levels. In this model, income level was also specified to predict PTSS and paternal involvement, and PTSS was specified to predict harsh and inconsistent discipline. We controlled for cancer stage and time since diagnosis. Results are shown in Figure 1 in the form of standardized beta coefficients. This model was saturated (i.e., *df* = 0).

As predicted, family income was associated with lower levels of paternal involvement, which was associated with greater PTSS in mothers. PTSS, in turn, were associated with more harsh and inconsistent discipline practices. The model explained 17% of the variance of paternal involvement, 34% of the variance of PTSS, and 22% of the variance of maternal harsh and inconsistent discipline. A more advanced stage (β = 0.21, *p* < 0.05) and less time since diagnosis (β = −0.24, *p* < 0.01) was also associated with lower PTSS. However, neither paternal involvement nor harsh and inconsistent discipline was significantly predicted by stage and time since diagnosis. The direct effect of family income on maternal harsh and inconsistent discipline, above and beyond paternal involvement, maternal PTSS, stage, and time since diagnosis, was not significant (see Figure 1).

Next, we tested the indirect, direct, and total effects of income level on harsh and inconsistent maternal discipline using Bayesian confidence intervals (see Table 2). Several indirect effects in the model emerged as significant. Specifically, the 95% confidence interval of the indirect path between income level and maternal PTSS through paternal involvement did not include 0, indicating significance at the *p* < 0.05 level. As predicted, a greater income level was associated with more paternal involvement, which in turn was associated with lower PTSS in mothers (β = −0.14, CI [−0.26, −0.05]). The indirect effect of paternal involvement on harsh and inconsistent discipline through PTSS was also significant (*p* < 0.05), such that greater paternal involvement was associated with lower PTSS in mothers, which in turn was associated with lower harsh and inconsistent maternal discipline (β = −0.14, CI [−0.28, −0.03]). Finally, the serial indirect effect of family income on the mother's harsh and inconsistent discipline through parental involvement and the mother's PTSS was significant at the *p* < 0.05 level. As anticipated, a higher income level was associated with more paternal involvement, which was associated with lower levels of maternal PTSS, which, in turn, were associated with lower harsh and inconsistent maternal discipline (β = −0.06, CI [−0.13, −0.01]). The total indirect effect of family income on harsh and inconsistent discipline, which includes all indirect paths, significant or non-significant, from family income to harsh and inconsistent discipline, was significant (β = −0.15, CI [−0.29, −0.04]). However, the total effect, which includes all direct and indirect paths from family income to harsh and inconsistent discipline, failed to reach significance, despite the significant zero-order correlation between these variables.

**Table 2:** Total, direct and indirect effects of income, paternal involvement, PTSS and maternal harsh and inconsistent discipline.

**Path**	**Coefficient**	**SE**	**Lower CI**	**Upper CI**
Income > PI > HID	−0.03	0.001	−0.14	0.07
Income > PTSS > HID	−0.05	0.001	−0.14	0.02
Income > PI > PTSS	**−0.14**	**0.001**	–**0.26**	–**0.05**
PI > PTSS > HID	–**0.14**	**0.001**	–**0.28**	–**0.03**
Income > PI > PTSS > HID	–**0.06**	**0.001**	–**0.13**	–**0.01**
Income > HID				
Total effect	−0.26	0.003	−0.45	0.05
Direct effect	−0.11	0.001	−0.32	0.15
Total indirect effect	–**0.15**	**0.001**	–**0.29**	–**0.04**

*PI, Paternal Involvement; PTSS, Post Traumatic Stress Symptoms; HID, Harsh and Inconsistent Discipline. Significant paths are bold*.

Because psychological distress may affect perceived support (45), we also tested an alternative model in which the order of the mediators was reversed (maternal PTSS predicting paternal involvement). This model did not yield a significant indirect effect.

## Discussion

BC is a potentially traumatic event (24) that may adversely affect maternal functioning [for a review, see (71)]. Relying on the FSM (16, 33) and COR theory (34), the current study aimed to examine a process by which promotive factors (i.e., economic resources and engagement of fathers in parenting) serially predict lower PTSS and in turn less harsh and inconsistent discipline in mothers coping with BC treatments. In line with our hypotheses and theory, we found a serial mediation process in which economic resources promote more paternal involvement, which was linked with lower maternal PTSS, and in turn, with less harsh and inconsistent discipline practices in mothers undergoing BC treatments.

The present study indicates that during BC treatments, having greater PTSS levels places mothers at risk for engagement in ineffective parenting practices. These findings support the FSM (16) and research showing that under stressful conditions, distressed parents tend to have more negative interchanges with their children (23, 33). The current study, however, expands the scope of the extant literature by focusing on the effect of PTSS on harsh and inconsistent discipline in the context of BC. Individuals with PTSS tend to display more anger and hostile behaviors (72). PTSS may deplete the mental resources of mothers, making it more difficult to tolerate intense mother-child interactions (73), which may lead to coercive and inconsistent patenting behaviors (16, 17). It should be noted that our study indicated that the effect of PTSS on maternal practices is above and beyond the severity of the disease and the time since it was diagnosed. Consistent with previous research, these findings highlight that the psychological response to trauma rather than the traumatic event itself is the main predictor of engagement in negative parental practices (74).

The study sheds light on a less studied parental behavior in the context of BC: harsh (e.g., yelling) and inconsistent discipline. Research on the parenting of mothers with BC is focused mostly on cancer-related parental tasks such as communicating about the disease (75). Harsh and coercive practices have been repeatedly found to predict behavioral problems in children and adolescents [for a review, see (33)]. Given that children of cancer-patient mothers are at risk to develop psychological and behavioral problems (12, 13), and given that parenting is a central protective factor for children exposed to adversity (14), more attention should be given to this topic.

The present study further points to a possible process of maternal resilience during BC treatments. Specifically, greater financial resources may be particularly important for fathers and promote their greater involvement in parenting tasks, which may then lead to better mental health and parenting outcomes in mothers. This finding suggests a chain of resource gain. The COR theory posits that existing resources may serve as a substrate for the gain or loss of other resources (34–36). In the context of BC, fathers and mothers are required to deal with multiple tasks, challenges, and changes [for a review, see (75)]. This may require out-of-pocket financial investments to cover mounting costs, in addition to caregiving support, often while losing income due to work reduction (39). When confronting uncertain stressful conditions, material resources facilitate a greater sense of security and better capacity to handle stress, thereby promoting better psychological adaptation (34, 36). Psychological security may, consequently, increase fathers' availability to engage in parenting tasks.

We found that in families with more financial resources, fathers were more likely to take part in parenting, which in turn, was associated with lower levels of PTSS in mothers. The COR theory (34–36) explains that having more resources facilitates successful coping with stress. Indeed, previous research indicated that having fewer economic resources predicts PTSD following exposure to trauma (76), including BC (29). Our study points to an indirect pathway by which economic resources promote better mental health in terms of PTSS, through paternal involvement. Social support has long been recognized as a protective factor against PTSD. However, while the extant research focused on general support (51, 52), the present study showed that support in the form of paternal involvement may be uniquely beneficial to the mental health of mothers during BC treatments.

While undergoing intensive cancer treatments with significant side effects, mothers may fail to maintain a family routine and perform their parental roles (77). BC is a condition in which mothers are required to shift their attention to their own recovery, which may disrupt day-to-day maternal functioning. Mothers are very often the primary caregivers of children and may struggle to balance their own and their children's needs [for a review, see (75)]. The inability to effectively carry out their maternal role is a major source of distress for mothers with cancer (78). For example, mothers are worried about the effects of their cancer on their children's wellbeing and feel guilt, shame, and loss of control [for a review, see (75)]. Thus, having a partner who takes an active part in parenting can facilitate a sense of security, a fundamental protective factor against PTSS when encountering threatening situations such as cancer (34).

Overall, our model suggests a cascading process by which economic resources facilitate a sequence of adaptive behaviors and coping, culminating in better maternal functioning when coping with BC. The current model adds to prior findings regarding the effects of economic hardship, trauma, social support, and PTSS on parenting [e.g., (20, 22, 23)] by examining a comprehensive process combining these factors. The model illuminates a promotive process for maternal functioning at the intersection between cancer and economic condition. Specifically, although cancer may be a stressful condition for any family, good financial status is a significant resource that may lead to a chain of reactions resulting in better paternal functioning and improved maternal functioning and mental state. On the other hand, the findings also suggest that a lack of sufficient financial resources places women with BC at risk of losing additional resources such as paternal involvement. Poor resources may signify the situation as more threatening and hence may intensify PTSS and, in turn, intensify harsh and inconsistent discipline.

### Limitations and Future Directions

Several limitations should be taken into consideration when interpreting the results of the current study. First, this is a cross-sectional, correlational study, and therefore no causality or chronological order can be inferred. Given the correlational design, it cannot be concluded that greater economic resources *cause* greater involvement of fathers and subsequently lower levels of PTSS and better maternal functioning. In addition, although an alternative model ruled out that maternal PTSS mediates the relationship between income level and paternal involvement, only a longitudinal design could delineate the temporal sequence of the variables examined in the model.

Second, the exclusive use of self-report measures may be vulnerable to various biases and statistical artifacts (e.g., self-presentation, common method variance). Thus, future studies should incorporate other types of measures and sources of information (e.g., observations, multi-informant surveys). Third, this study used a relatively small sample of 100 women recruited in closed social media groups for women with BC and included only Jewish Israeli women. Therefore, it may not be representative of the entire population of women dealing with BC. It is important to replicate the model in more culturally diverse populations where gender roles in parental tasks may differ. Another issue concerning the generalizability of the findings is related to the specific social policy in Israel. Israel provides universal public health services. Cancer patients also qualify for social security benefits and supportive psychosocial interventions for the target patient and the family without out-of-pocket costs. Perhaps the support provided by the social and healthcare systems in Israel weakened the effects in the model, which accounted for only 22% of the variance in harsh and inconsistent discipline. More research is needed in diverse societal contexts, for instance, in societies that do not have effective universal social and healthcare systems. Another limitation is related to the relatively low reliability of the harsh and inconsistent measures. This finding is consistent with previous findings describing the psychometric properties of the APQ (64). However, the low alpha coefficient may have reduced the effect sizes in the model. Finally, the present study examined family income level as an indicator of family economic resources. Future research should examine the effect of economic recourses in a more detailed way, for example, by testing the serial effect of being above vs. below the poverty line on fathers' and mothers' functioning. These findings may broaden the understanding of the impact of family economic resources on families dealing with BC.

### Clinical Implications

A BC diagnosis is often perceived by mothers as the most distressing event in their lives (79), and brings about a range of challenges that may alter mother-child relationships (71). Beyond the physical and emotional difficulties experienced during cancer treatments, a cancer diagnosis may undermine mothers' identity as effective parents [for a review, see (75)]. It is therefore imperative to develop interventions designed to the unique needs of mothers with BC. The present study points to possible promotive resources against PTSS and harsh and inconsistent discipline in mothers coping with BC. Our findings suggest that interventions should incorporate a systemic approach when assisting families coping with BC. At the societal level, interventions should focus on providing financial aid (e.g., disability benefits). At the family level, the model suggests that family interventions aimed at strengthening paternal functioning and co-parenting relationships may sequentially lead to better adaptation of mothers coping with BC. At the individual level, interventions designed to prevent post-traumatic distress may help mothers in their maternal role and ultimately promote their children's wellbeing.

## Conclusion

BC is a stressful condition that may adversely impact mental health and parental functioning in mothers. This study examined a process by which resource gain is linked with more negative maternal practices during BC treatments. In line with the FSM and COR theory, the study highlights a cascading process by which, when coping with BC, having more financial resources may promote more paternal involvement, which may lead to lower maternal PTSS levels and, in turn, to less harsh and inconsistent maternal discipline. This is important because such harsh and inconsistent parenting practices have been implicated in the development of children's psychological and behavioral problems.

## Data Availability Statement

The original contributions presented in the study are included in the article/supplementary materials, further inquiries can be directed to the corresponding author/s.

## Ethics Statement

The studies involving human participants were reviewed and approved by Hebrew University of Jerusalem. The patients/participants provided their written informed consent to participate in this study.

## Author Contributions

OZ is the principal investigator; she contributed to the research design, collection and analysis of the data, and writing all the parts of the manuscript. GB and YH contributed to the conceptualization of the model. All authors contributed to the article and approved the submitted version.

## Conflict of Interest

The authors declare that the research was conducted in the absence of any commercial or financial relationships that could be construed as a potential conflict of interest.

## Publisher's Note

All claims expressed in this article are solely those of the authors and do not necessarily represent those of their affiliated organizations, or those of the publisher, the editors and the reviewers. Any product that may be evaluated in this article, or claim that may be made by its manufacturer, is not guaranteed or endorsed by the publisher.
